# Triphenylphosphine-based functional porous polymer as an efficient heterogeneous catalyst for the synthesis of cyclic carbonates from CO_2_

**DOI:** 10.1186/s11671-017-2376-2

**Published:** 2017-11-28

**Authors:** Siduo Wu, Chao Teng, Sheng Cai, Biwang Jiang, Yong Wang, Hong Meng, Huchun Tao

**Affiliations:** 1 Guangdong Provincial Key Laboratory of Nano-Micro Material Research, School of Chemical Biology & Biotechnology, Peking University Shenzhen Graduate School, Shenzhen, 518055 China; 2 State Key Laboratory of Coordination Chemistry, School of Chemistry and Chemical Engineering, Nanjing University, Nanjing, 210093 China; 3 School of Advanced Materials, Peking University Shenzhen Graduate School, Shenzhen, 518055 China; 4 Key Laboratory for Heavy Metal Pollution Control and Reutilization, School of Environment and Energy, Peking University Shenzhen Graduate School, Shenzhen, 518055 China

**Keywords:** Porous polymer, Heterogeneous catalyst, Fixation of CO_2_, Cyclic carbonates

## Abstract

A novel triphenylphosphine-based porous polymer (TPDB) with a high Brunauer–Emmett–Teller (BET) surface area was synthesized through Friedel–Crafts alkylation of triphenylphosphine and α-dibromo-*p*-xylene. Then, the functional hydroxyl groups were successfully grafted onto the polymer framework by post modification of TPDB with 3-bromo-1-propanol (BP) and triethanolamine (TEA). The resulting sample TPDB-BP-TEA was characterized by various techniques such as FT-IR, TG, SEM, EDS mapping, ICP-MS, and N_2_ adsorption–desorption. This new polymer was tested as the catalyst in the solvent-free cycloaddition reaction of CO_2_ with epoxides, which exhibited excellent performance, with high yield, selectivity, and stable recyclability for several catalytic cycles. The comparison experiment results demonstrate that the bromide ions and hydroxyl groups, as well as high surface area, are key factors in improving the catalytic activity of this new catalyst.

**Electronic supplementary material:**

The online version of this article (10.1186/s11671-017-2376-2) contains supplementary material, which is available to authorized users.

## Background

Ionic liquids (ILs) have been attracted significant attention as alternative reaction media/catalysts because of their specific properties, such as negligible volatility, excellent thermal stability, remarkable solubility, and the variety of structures [1–3]. Particularly, ILs could be designed and modified with various functional groups in their cations or anions to gain the functionalities required by target reactions [4, 5]. Many IL-catalyzed organic reactions have been reported, among which cycloaddition reactions are a hot topic [6, 7]. Since carbon dioxide (CO_2_) is a potentially abundant, cheap, non-toxic, nonflammable, and renewable carbon resource in organic synthesis, great effort has been made to develop effective processes for CO_2_ chemical fixation. Recently, the cycloaddition of CO_2_ with epoxides for the synthesis of valuable cyclic carbonates is expected to be one of the most promising strategies for effective fixation of CO_2_ [8–11]. The products cyclic carbonates have found extensive applications as aprotic solvents, precursors, fuel additives, and green reagents. Though ILs have demonstrated to be excellent catalysts for the cycloaddition of CO_2_ at metal-free/solvent-free conditions, these homogeneous catalysts inevitably suffered from some problems of catalyst recovery and product purification.

The porous materials with high surface area open up new possibilities for the design and synthesis of new heterogeneous catalysts [12–14]. During the last few decades, in addition to traditional porous zeolites and activated carbon, a number of useful porous materials such as metal organic frameworks (MOFs) [15, 16], covalent organic frameworks (COFs) [17, 18], and porous organic polymers [19, 20] were developed and applied as catalyst supports for heterogeneous catalysis. Among these porous materials, IL-containing porous organic polymers have attracted particular attention due to their low skeletal density, high chemical stability, and the capability of introducing a broad range of useful functional groups within the porous framework [21–23]. For example, He et al. have developed a series of novel heterogeneous catalyst by immobilizing imidazolium-based ILs on an FDU-type mesoporous polymer, which show a good catalytic activity in the CO_2_ cycloaddition reaction [24]. However, multistep IL-modification method will inevitably suffer from the low IL loading amount and the inhomogeneous distribution of ILs. Besides the post modification strategy, direct synthesis of IL-containing polymer by radical polymerization is an alternative approach. For example, Wang and co-workers reported a template-free radical self-polymerization method to synthesize a mesoporous hierarchical poly(ionic liquid)s [25]. The obtained poly (ionic liquid)s present high activity, easy recycling, and reuse in the cycloaddition of CO_2_. Although various ionic polymers with abundant functional species can be obtained, the high BET surface area and high IL loading amount still cannot be acquired simultaneously make this copolymerization technique embarrassing. Therefore, the incorporation of IL groups into porous organic polymer framework with a high stable content and large surface area is still a great challenge.

In this paper, we reported the synthesis of triphenylphosphine-based ionic porous polymer with high surface area, large pore volume, and abundant bromide ions and hydroxyl groups for the cycloaddition of CO_2_ with epoxides. First, triphenylphosphine (PPh_3_) and α-dibromo-*p*-xylene (DB) were reacted to form porous polymer (TPDB) through Friedel–Crafts alkylation with anhydrous FeCl_3_ as a promoter. Then, the TPDB can be easily functionalized by 3-bromo-1-propanol (BP) and triethanolamine (TEA), respectively, to afford functional porous polymer (TPDB-BP-TEA). TPDB-BP-TEA was characterized by employing FTIR, TG, SEM, EDS mapping, ICP-MS, and N_2_ adsorption–desorption. Systematic catalytic tests show that the porous polymer is excellent catalyst for cycloaddition of CO_2_ to epoxides, with the advantages of high activity and selectivity, easy recovery, and steady reuse.

## Experimental

### Materials and methods

All the chemicals were of chemical grade and used as purchased. Thermogravimetry (TG) analysis was conducted with a STA409 instrument at a heating rate of 10 K/min in nitrogen. Fourier-transform infrared (FT-IR) spectra were recorded on an Agilent Cary 660 FT-IR spectrometer in the 4000–400 cm^−1^ region with the tested samples pressed into KBr disks. Scanning electron microscopy (SEM) images were recorded on a SUPERSCAN SSX-550 electron microscope (Shimadz, Japan) operating at 20 kV. The phosphorus (P), oxygen (O), and nitrogen (N) element distribution were characterized by Hitachi S-4800 field emission scanning electron microscope accompanied by energy dispersive X-ray spectrometry. BELSORP-MINI instrument was used to measure the nitrogen sorption-isotherms at liquid nitrogen (77 K) temperature. The specific surface areas were evaluated using the Brunauer–Emmett–Teller (BET) method, and the pore distribution was calculated by the BJH method from adsorption branches of isotherms. The P element content was determined by ICP-MS using Agilent 7700 spectrometer. CHN elemental analysis was performed on an elemental analyzer Vario EL cube.

### Catalyst preparation

#### Synthesis of TPDB

TPDB was prepared according to the previous literature [26]. PPh_3_ (4 mmol, 1.05 g) and α-dibromo-*p*-xylene (DB, 4 mmol, 1.06 g) were dissolved in 20 mL 1,2-dichloroethane (DCE). Then, anhydrous FeCl_3_ (16 mmol, 2.59 g) was added in the above solution to catalyze the alkylation between PPh_3_ and DB. The reaction mixture was first stirred at 45 °C for 5 h and then reacted at 80 °C for another 48 h. On completion, the resulting brown gel was filtered out and Soxhlet extracted with DCE and methanol for 24 h, respectively. The cross-linked polymer TPDB was obtained after drying at 60 °C under vacuum condition.

#### Synthesis of TPDB-BP

The obtained polymer TPDB (1 g) was dispersed in 15 mL acetonitrile, and 3-bromo-1-propanol (BP, 0.8 g) was added into the solution. The reaction mixture was reacted at 80 °C for 24 h. The solid product TPDB-BP was filtered, washed with acetonitrile for three times, and dried at 60 °C under vacuum condition.

#### Synthesis of TPDB-BP-TEA

TPDB-BP (1 g) was dispersed in 15 mL acetone, and then, triethanolamine (TEA, 0.8 g) was added into it. The reaction mixture was reacted at 60 °C for 24 h. On completion, the solid product TPDB-BP-TEA was filtered and washed with acetone for three times, followed by drying in vacuum at 60 °C for 12 h. ICP-MS analysis result disclosed 3.7 wt% of P element within TPDB. CHN elemental analysis results found (wt%) C 64.91%, H 5.54%, and N 1.65%.

### Catalytic test

The cycloaddition reaction was performed in a stainless steel autoclave reactor (25 mL) with a magnetic stirrer. In a typical run, propylene oxide (PO, 20 mmol) and catalyst TPDB-BP-TEA (0.1 g) were placed in the autoclave reactor. CO_2_ was then charged to 1 MPa, and the reaction temperature was adjusted to 120 °C. The reaction mixture was reacted for 6 h, after which, the reactor was cooled to ambient temperature, and ethyl alcohol was added into it to dilute the reaction mixture. The solid catalyst was filtered out, and the filtrate was analyzed by gas chromatography (GC) using biphenyl as an internal standard to calculate the yield. GC was equipped with a FID and a DB-wax capillary column (SE-54 30 m × 0.32 mm × 0.25 μm). The GC spectra are shown in Additional file 1: Figures S1–S5.

## Results and discussion

### Synthesis and characterization of catalysts

**Scheme 1:**
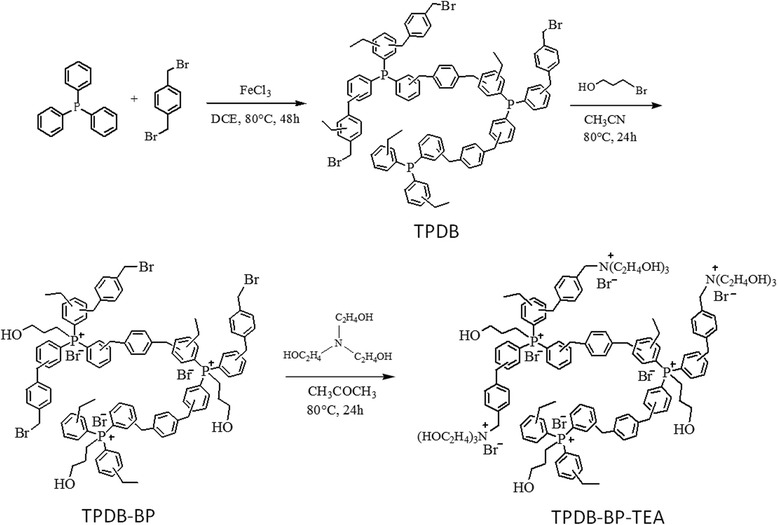
Synthesis of porous organic polymer TPDB-BP-TEA. First, triphenylphosphine (PPh_3_) and α-dibromo-*p*-xylene (DB) were reacted to form porous polymer (TPDB) through Friedel–Crafts alkylation with anhydrous FeCl_3_ as a promoter. Then, the TPDB can be easily functionalized by 3-bromo-1-propanol (BP) and triethanolamine (TEA), to afford functional porous polymer (TPDB-BP-TEA)

**Fig. 1:**
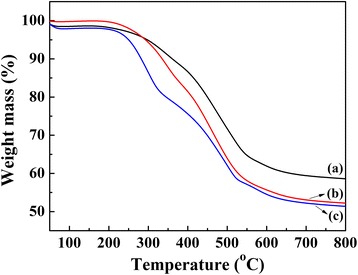
TG curves of (a) TPDB, **(**b**)** TPDB-BP, and **(**c) TPDB-BP-TEA. TPDB was found to be stable up to 300 °C as evidenced by TG (curve a). After the modification with BP and TEA, the thermos stability of the obtained samples TPDB-BP and TPDB-BP-TEA slightly decreased to 250 °C

According to the synthesis procedure illustrated in Scheme 1, a porous organic polymer TPDB was prepared by Friedel–Crafts alkylation of PPh_3_ by using DB as a cross-linker and FeCl_3_ as a promoter. TPDB was then functionalized with BP, affording the functionalized polymer TPDB-BP. Further modification of TPDB-BP with TEA gave the resulting TPDB-BP-TEA, which was thoroughly characterized by TG, FT-IR, SEM, EDX, and N_2_ adsorption/desorption analysis. TPDB was found to be stable up to ca. 300 °C as evidenced by TG (Fig. 1, curve a). After the modification with BP and TEA, the thermostability of the obtained samples TPDB-BP and TPDB-BP-TEA slightly decreased to 250 °C (Fig. 1, curves b and c). ICP-MS analysis result disclosed 3.7 wt% of P element within TPDB-BP-TEA, and CHN elemental analysis shows C 64.91 wt%, H 5.54 wt%, and N 1.65 wt% for TPDB-BP-TEA.

**Fig. 2:**
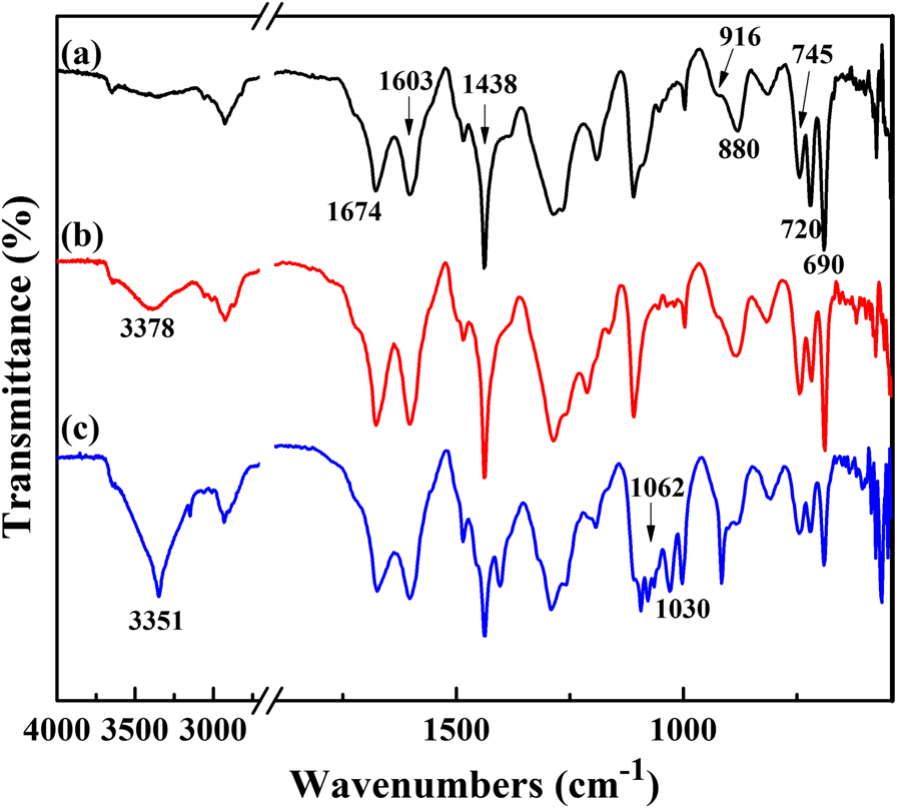
FT-IR spectra of (a) TPDB, (b) TPDB-BP, and (c) TPDB-BP-TEA. The distinct bands corresponding to the P–C=C (1674 cm^−1^) in PPh_3_ and aromatic ring stretching vibrations (1603–1438 cm^−1^), as well as to the stretching vibrations of C−H in aromatic ring (916, 880, 745, 720, and 690 cm^−1^) indicates the presence of both PPh_3_ and DB groups in TPDB. TPDB-PA shows a moderate intensity broad absorption band at 3378 cm^−1^, which is corresponding to the stretching vibration of the –OH. After further modified by TEA, the intensity of –OH vibration at 3351 cm^−1^ for TPDB-BP-TEA significantly increased. Besides, the new bands appeared at 1062 and 1030 cm^−1^ are assigned to the stretching vibrations of C–N and C–O in TEA, respectively

**Fig. 3:**
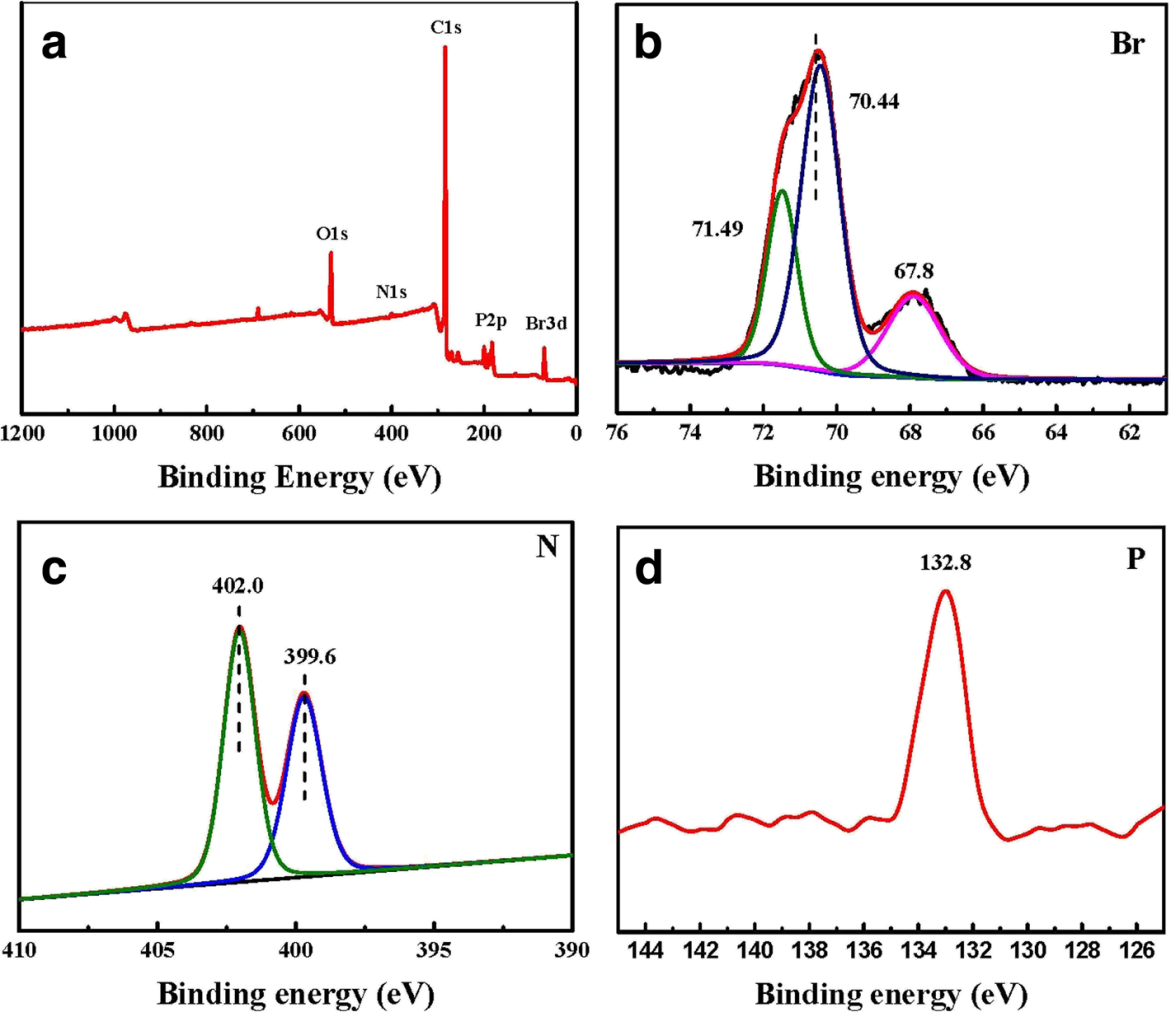
**a** Wide XPS spectrum, **b** Br spectrum, **c** N spectrum, and **d** P spectrum of TPDB-BP-TEA. The wide XPS spectra in Fig. 3 indicate the presence of P, C, N, Br, and O elements on TPDB-BP-TEA

Figure 2 shows the FT-IR spectra of TPDB polymer and after its stepwise modification. The distinct bands corresponding to the P–C=C (1674 cm^−1^) in PPh_3_ and aromatic ring stretching vibrations (1603–1438 cm^−1^), as well as to the stretching vibrations of C−H in aromatic ring (916, 880, 745, 720, and 690 cm^−1^) are present in the FT-IR spectrum of TPDB, indicating the present of both PPh_3_ and DB groups. After the modification of BP, the observed bands are similar. However, TPDB-PA shows a moderate intensity broad absorption band at 3378 cm^−1^, which is corresponding to the stretching vibration of the –OH. After TPDB-BP was further modified by TEA, the intensity of –OH vibration at 3351 cm^−1^ for TPDB-BP-TEA significantly increased, which is probably due to the effect of the abundant organic groups (−N(CH2CH2OH)_3_). Besides, the new bands appeared at 1062 and 1030 cm^−1^ are assigned to the stretching vibrations of C–N and C–O in TEA, respectively. The wide XPS spectra in Fig. 3 indicate the presence of P, C, N, Br, and O elements on TPDB-BP-TEA. These observations suggest that the BP and TEA groups were successfully grafted on the framework of TPDB.

**Fig. 4:**
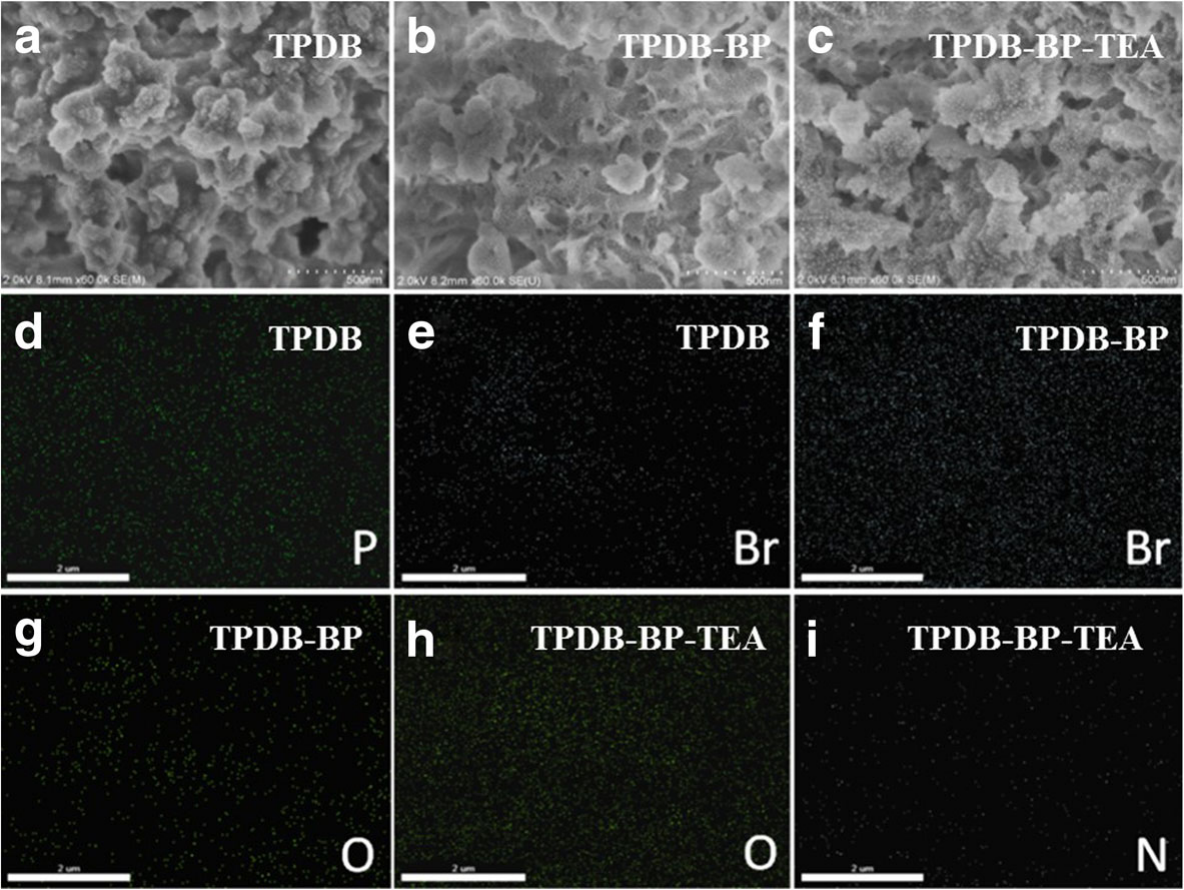
SEM and EDS mapping images of **a**, **d**, and **e** TPDB, **b**, **f**, and **g** TPDB-BP, and **c**, **h**, and **i** TPDB-BP-TEA. TPDB, TPDB-BO, and TPDB-BP-TEA all show amorphous morphology (**a**). After the modification with BP and TEA, TPDB-BP-TEA shows no main changes in the structure, but its surface became rough with some agglomerated blocks (**c**). EDS mapping image validates the homogeneous distribution of P and Br elements in the polymer framework of TPDB (**d**, **e**). The amount of Br increased obviously (**f**), and a new element O was observed (**g**) after the modification of TPDB with BP. After the further modification of TPDB-BP with TEA, a new element N was observed (**i**), and the amount of O element increased significantly in the image of TPDB-BP-TEA (**h**). These images confirm the successful immobilization of BP and TEA on the TPDB framework

The samples were further characterized by SEM and EDS mapping (Fig. 4). TPDB shows an amorphous morphology (Fig. 4a). When the BP was tethered onto the TPDB framework, TPDB-BP also presents the amorphous morphology with nanoscale hollow structure (Fig. 4b). After the further modification with TEA, TPDB-BP-TEA shows no main changes in the structure, but its surface became rough with some agglomerated blocks (Fig. 4c). EDS mapping image validates the homogeneous distribution of P and Br elements in the polymer framework of TPDB (Fig. 4d, e). The amount of Br increased obviously (Fig. 4f), and a new element O was observed (Fig. 4g) after the modification of TPDB with BP. After the further modification of TPDB-BP with TEA, a new element N was observed (Fig. 4i), and the amount of O element increased significantly in the image of TPDB-BP-TEA (Fig. 4h). These images confirm the successful immobilization of BP and TEA on the TPDB framework, which was in agreement with the FT-IR analysis.

**Fig. 5:**
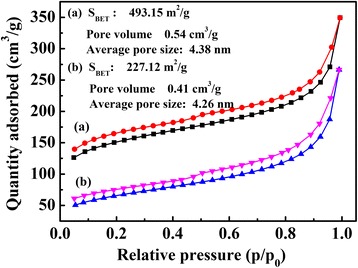
Nitrogen adsorption–desorption isotherms of (a) TPDB and (b) TPDB-BP-TEA. BET surface areas and pore size distributions of the polymers TPDB and TPDB-BP-TEA were measured by analyzing N_2_ adsorption and desorption isotherms at 77 K. The steep rise in the high P/P_0_ region indicates that the material consists of micropores and mesopores. TPDB presents a high BET surface area of 493.15 m^2^/g, pore volume of 0.54 cm^3^/g, and average pore size of 4.38 nm. After the two-step modification, the BET surface area and pore volume decreased to 227.12 m^2^/g and 0.41 cm^3^/g, respectively

BET surface areas and pore size distributions of the polymers TPDB and TPDB-BP-TEA were measured by analyzing N_2_ adsorption and desorption isotherms at 77 K. As shown in Fig. 5, TPDB and TPDB-BP-TEA show an initial high uptake, followed by a gradual increase in nitrogen adsorption, and the steep rise in the high P/P_0_ region indicates that the material consists of micropores and mesopores. TPDB presents a high BET surface area of 493.15 m^2^/g, pore volume of 0.54 cm^3^/g, and average pore size of 4.38 nm. After the two-step modification, the BET surface area and pore volume decreased to 227.12 m^2^/g and 0.41 cm^3^/g, respectively. The decrease of surface area and pore size was probably due to that the modification process has led to slightly loss of pore efficacy, while the integral pore structure of the catalyst remains unchanged.

### Catalytic performance of catalysts

The catalytic performance of all samples was first evaluated by performing the cycloaddition of CO_2_ and PO under mild conditions without the aid of any metal, solvent, and external homogeneous co-catalyst, and the results are shown in Table 1. Initially, no cyclic carbonate product was detected without using any catalyst (entry 1). When TPDB was used as the catalyst, 44% yield of cyclic carbonate with a low selectivity of 59% was observed and 1,2-propanediol is formed as major byproduct (entry 2). TPDB-BP exhibited a slightly increased yield of 51% with 93% selectivity (entry 3). After the further modification with TEA, interestingly, TPDB-BP-TEA offered a very high yield of 97% with 100% selectivity (entry 4). It is well known in the literatures that Br anions act as the main active centers for the cycloaddition reactions [27–30]. Moreover, the presence of –OH groups on solid materials could efficiently promote the ring opening of epoxides due to hydrogen bonding [31–33]. Therefore, the excellent performance of TPDB-BP-TEA is reasonably related to its abundant Br anions and –OH groups as suggested by EDS mapping in Fig. 4f. Additionally, the high surface and porous structure of the catalyst can in principle accelerate the interfacial mass and energy transfer.

**Table 1:** Cycloaddition of CO_2_ and PO catalyzed by various catalysts

Entry	Catalyst	Solubility	Yield^a^ (%)	Sel^b^ (%)
1	No catalyst	Homogeneous	–	–
2	TPDB	Heterogeneous	44	59
3	TPDB-BP	Heterogeneous	51	93
4	TPDB-BP-TEA	Heterogeneous	97	100

Reaction conditions: PO (20 mmol), CO_2_ (1.0 MPa), catalyst (0.10 g), 120 °C, 4 h

^a^The yield of cyclic carbonate product

^b^The selectivity for the cyclic carbonate product, the byproduct is mostly 1,2-propanediol

**Fig. 6:**
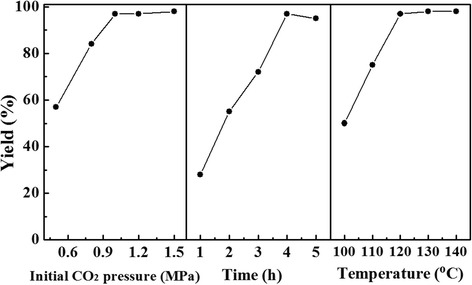
Influence of reaction parameters on the cycloaddition reaction of CO_2_ with propylene oxide. The yield remarkably increased from 58 to 97% when the CO_2_ pressure was increased from 0.6 to 1.0 MPa and after that the yield maintained constant. The catalytic reaction finished in 4 h, whereas longer reaction time caused a slightly decrease of yield. This is maybe due to the side reactions like polymerization of PC. The optimal reaction temperature was 120 °C

The influence of the reaction parameters, such as initial CO_2_ pressure, reaction time, and temperature, was investigated using TPDB-BP-TEA as the catalyst, and the results are summarized in Fig. 6. The yield remarkably increased from 58 to 97% when the CO_2_ pressure was increased from 0.6 to 1.0 MPa and after that the yield maintained constant. The catalytic reaction finished in 4 h, whereas longer reaction time caused a slightly decrease of yield. This is maybe due to the side reactions like polymerization of PC. Moreover, a reaction temperature of 120 °C was optimal for the synthesis of cyclic carbonate in this study. Apart from PO, TPDB-BP-TEA exhibits highly efficient activity for cycloaddition of various epoxides (Table 2), including the epichlorohydrin, allyl glycidyl ether, and styrene oxide (GC spectra are shown in Additional file 1: Figures S1–S5). As very challenging substrates for this reaction, internal epoxides require drastic conditions for efficient conversion due to that “apparent” size selective catalysis is obvious in porous heterogeneous systems [34–36]. Herein, cyclohexene oxide shows a relatively low yield of 74% with 59% selectivity over porous TPDB-BP-TEA catalyst, which is probably because of the inherent inertness of cyclohexene oxide.

**Table 2:** Cycloaddition of CO_2_ to different epoxides catalyzed by TPDB-BP-TEA

Entry	Epoxide	Product	Time (h)	Con (%)	Sel (%)
1			4	97	> 99
2			4	95	95
3			4	96	98
4			6	84	> 99
5			16	74	59

Reaction conditions: epoxides 20 mmol, catalyst TPDB-BP-TEA 0.10 g, temperature 120 °C, initial CO_2_ pressure 1.0 MPa

**Fig. 7:**
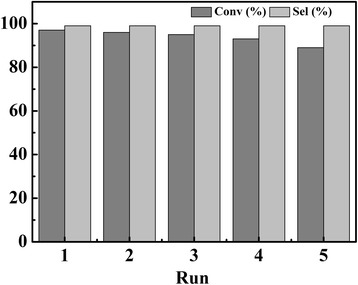
Catalytic reusability of TPDB-BP-TEA for cycloaddition of CO_2_ with PO. As a solid catalyst, TPDB-BP-TEA is recovered readily by filtration or centrifugation and well maintain its activity in the five-run recycling test under mild conditions

**Fig. 8:**
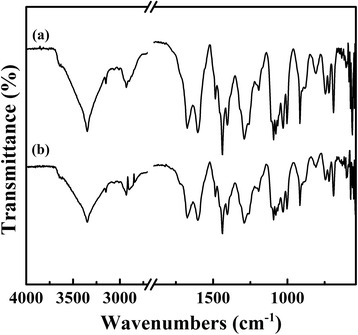
FT-IR spectra of (a) fresh TPDB-BP-TEA and (b) reused TPDB-BP-TEA. FT-IR spectrum of the recovered catalyst suggests the well-preserved textural properties relative to the fresh one, accounting for its well recyclability

As is depicted in Fig. 7, TPDB-BP-TEA is recovered readily by filtration or centrifugation and well maintains its activity in the five-run recycling test under mild conditions. The reaction conditions are the same with that in Table 1. In order to verify the leaching of the catalyst, a hot-filtration experiment was further carried out. After the TPDB-BP-TEA catalyst was removed from the reaction solution after 2 h (yield 59%), the supernatant did not show any further reactivity over the next 4 h, indicating the heterogeneous nature of the present catalyst. ICP-MS and CHN analyses for the recycled catalyst show 3.84 wt% P, 67.72 wt% C, 5.83 wt% H, and 1.52 wt% N, which are very similar to the fresh catalyst. FT-IR spectrum of the recovered catalyst (Fig. 8) suggests the well-preserved textural properties relative to the fresh one, accounting for its well recyclability.

## Conclusions

A porous organic polymer with large surface area, high density of ionic sites, and functional –OH groups is developed by Friedel–Crafts alkylation and post modification reaction. The resulting sample TPDB-BP-TEA could be used as the highly efficient heterogeneous catalyst for the synthesis of cyclic carbonates from cycloaddition of CO_2_ and epoxides under metal-free and solvent-free conditions. Relative high yields and selectivity are obtained over various substrates, and the catalyst can be facilely separated and reused with very steady activity. The abundant bromide ions and hydroxyl groups, the porous structure, and high surface area are revealed to be responsible for the catalyst’s excellent performances in cycloaddition of CO_2_. The approach in this work triggers an ideal pathway for an easy access to a series of porous, functionalizable polymers, which not only can be applied for chemical fixation of CO_2_ into fine chemicals, but is also promising for a myriad of potential catalytic applications.

## Additional file

Additional file 1: Figure S1. GC spectrum for the cycloaddition reaction of CO_2_ with propylene oxide over TPDB-BP-TEA. Figure S2. GC spectrum for the cycloaddition reaction of CO_2_ with epichlorohydrin over TPDB-BP-TEA. **Figure S3.** GC spectrum for the cycloaddition reaction of CO_2_ with allyl glycidyl ether over TPDB-BP-TEA. **Figure S4.** GC spectrum for the cycloaddition reaction of CO_2_ with styrene oxide over TPDB-BP-TEA. **Figure S5.** GC spectrum for the cycloaddition reaction of CO_2_ with cyclohexene oxide over TPDB-BP-TEA. (DOCX 598 kb)
