# Supplementary material for: Triphenylphosphine-based functional porous polymer as an efficient heterogeneous catalyst for the synthesis of cyclic carbonates from CO2
Source: Nanoscale Res Lett. 2017 Nov 28;12:609. doi: 10.1186/s11671-017-2376-2 (PMC5705529; doi:10.1186/s11671-017-2376-2)
Supplement: Additional file 1: Figure S1. — GC spectrum for the cycloaddition reaction of CO2 with propylene oxide over TPDB-BP-TEA. Figure S2. GC spectrum for the cycloaddition reaction of CO2 with epichlorohydrin over TPDB-BP-TEA. Figure S3. GC spectrum for the cycloaddition reaction of CO2 with allyl glycidyl ether over TPDB-BP-TEA. Figure S4. GC spectrum for the cycloaddition reaction of CO2 with styrene oxide over TPDB-BP-TEA. Figure S5. GC spectrum for the cycloaddition reaction of CO2 with cyclohexene oxide over TPDB-BP-TEA. (DOCX 598 kb) [file 11671_2017_2376_MOESM1_ESM.docx]

**Additional file 1**

Figure S1. GC spectrum for the cycloaddition reaction of CO_2_ with propylene oxide over TPDB-BP-TEA.

Figure S2. GC spectrum for the cycloaddition reaction of CO_2_ with epichlorohydrin over TPDB-BP-TEA.

Figure S3. GC spectrum for the cycloaddition reaction of CO_2_ with allyl glycidyl ether over TPDB-BP-TEA.

Figure S4. GC spectrum for the cycloaddition reaction of CO_2_ with styrene oxide over TPDB-BP-TEA.

Figure S5. GC spectrum for the cycloaddition reaction of CO_2_ with cyclohexene oxide over TPDB-BP-TEA.
